# Indoor residual spraying for kala-azar vector control in Bangladesh: A continuing challenge

**DOI:** 10.1371/journal.pntd.0006846

**Published:** 2018-10-01

**Authors:** Rajib Chowdhury, Vashkar Chowdhury, Shyla Faria, Saiful Islam, Narayan Prosad Maheswary, Shireen Akhter, Md. Sahidul Islam, Aditya Prasad Dash, Axel Kroeger, Qamar Banu

**Affiliations:** 1 International Centre for Diarrhoea Disease Research, Bangladesh (icddr,b), Dhaka, Bangladesh; 2 National Institute of Preventive and Social Medicine (NIPSOM), Mohakhali, Dhaka, Bangladesh; 3 Department of Statistics, Dhaka College, Dhaka, Bangladesh; 4 Central University of Tamil Nadu, Thiruvarur, India; 5 Special Programme for Research and Training in Tropical Diseases, World Health Organization, Geneva, Switzerland; 6 University of Freiburg, Centre for Medicine and Society/Anthropology, Freiburg, Germany; 7 Asian University for Women, Dampara, Chittagong, Bangladesh; RTI International, UNITED STATES

## Abstract

**Background:**

Visceral leishmaniasis (VL) in the Indian subcontinent is a fatal disease if left untreated. Between 1994 to 2013, the Ministry of Health of Bangladesh reported 1,09,266 cases of VL and 329 VL related deaths in 37 endemic districts. Indoor residual spraying (IRS) using dichlorodiphenyltrichloroethane (DDT) was used by the national programme in the 1960s to control malaria. Despite findings of research trials demonstrating that the synthetic pyrethroid deltamethrin 5 WP was very effective at reducing vector densities, no national VL vector control operations took place in Bangladesh between 1999 to early 2012. In 2012, IRS using deltamethrin 5 WP was re-introduced by the national programme, which consisted of pre-monsoon spraying in eight highly endemic sub-districts (upazilas). The present study aims to evaluate the effectiveness of IRS on VL vectors, as well as the process and performance of the spraying activities by national programme staff.

**Methods:**

Five highly endemic upazilas of Mymensingh district were purposively selected (Fulbaria, Trishal, Mukthagacha, Gaforgaon and Bhaluka) to conduct the present study using the WHO/TDR monitoring and evaluation tool kit. IRS operations, conducted by 136 squads/teams, and 544 spraymen, were observed using check lists and questionnaires included in the WHO/TDR monitoring and evaluation tool kit. A household (HH) acceptability survey of IRS was conducted in all study areas using a structured questionnaire in 600 HHs. To measure the efficacy of IRS, pre-IRS (two weeks prior) and post-IRS (at one and five months after), vector density was measured using CDC light traps for two consecutive nights. Bioassays, using the WHO cone-method, were carried out in 80 HHs (40 sprayed and 40 unsprayed) to measure the effectiveness of the insecticide on sprayed surfaces.

**Results:**

Of the 544 spraymen interviewed pre-IRS, 60%, 3% and 37% had received training for one, two and three days respectively. During spraying activities, 64% of the spraying squads had a supervisor in 4 upazilas but only one upazila (Mukthagacha) achieved 100% supervision of squads. Overall, 72.8% of the spraying squads in the study upazilas had informed HHs members to prepare their houses prior to spraying. The required personal protective equipment was not provided by the national programme during our observations and the spraying techniques used by all sprayers were sub-standard compared to the standard procedure mentioned in the M&E toolkit. In the HH interviews, 94.8% of the 600 respondents said that all their living rooms and cattle sheds had been sprayed. Regarding the effectiveness measurements (i.e. reduction of vector densities), a total of 4132 sand flies were trapped in three intervals, of which 3310 (80.1%) were *P*. *argentipes*; 46.5% (1540) males and 53.5% (1770) females. At one month post-IRS, *P*. *argentipes* densities were reduced by 22.5% but the 5 months post-IRS reduction was only 6.4% for both male and female. The bioassay tests showed a mean corrected mortality of *P*. *argentipes* sand flies at one month post-IRS of 87.3% which dropped to 74.5% at 4 months post-IRS in three upazilas, which is below the WHO threshold level (80%).

**Conclusion:**

The national programme should conduct monitoring and evaluation activities to ensure high quality of IRS operations as a pre-condition for achieving a fast and sustained reduction in vector densities. This will continue to be important during the maintenance phase of VL elimination on the Indian subcontinent. Further research is needed to determine other suitable vector control option(s) when the case numbers are very low.

## Author summary

The visceral leishmaniasis (VL) elimination programme was launched in the Indian subcontinent (Bangladesh, India and Nepal) in 2005. Although the integrated vector management (IVM) system is one of the important elements highlighted in the Regional VL elimination strategy, indoor residual spraying (IRS) is the sole intervention practice that has been implemented. In fact, in Bangladesh from 1999 to early 2012, no VL vector control was used at all and pre-monsoon IRS was only re-introduced by the national programme in eight high endemic upazilas (sub-districts) in 2012. The present study monitored IRS operation in five upazilas (Fulbaria, Trishal, Mukthagacha, Gaforgaon and Bhaluka). Monitoring took place with the help of using observation check lists and questionnaires included in the WHO/TDR monitoring and evaluation tool kit. The study identified that training of spraymen was insufficient and a supervisor was not always present during spraying. The spraying techniques by all the sprayers were sub-standard. It was also found that all the required personal protective equipment was not provided by the national programme. It is recommended that the national programme should conduct monitoring and evaluation activities to ensure high quality of IRS operations in order to achieve maximum benefit.

## Introduction

Visceral leishmaniasis (VL), also known as kala-azar (KA) in the Indian subcontinent (ISC), is a fatal disease if left untreated [1]. In the ISC, the disease is transmitted exclusively by the sand fly vector *Phlebotomus argentipes* [2]. *Leishmania donovani* is the protozoan causative agent. Visceral leishmaniasis is affecting marginalized communities worldwide [1,3]. Within the ISC, VL is highly prevalent in Bangladesh, India and Nepal; and some sporadic cases were reported from Bhutan [4]. Among the global burden, 90% of all VL cases are reported by only six countries (India, Bangladesh, Sudan, South Sudan, Ethiopia and Brazil) and it is estimated that 200,000 to 400,000 cases and 20,000 to 40,000 deaths occur annually on a global scal [3]. The first historical report of VL was from Jessore district, currently located in the south-western part of Bangladesh, where an epidemic outbreak killed an estimated 75,000 people between 1824 and 1827 [5]. In the 1960s, a comprehensive malaria eradication programme (MEP) was launched using dichlorodiphenyltrichloroethane (DDT) for indoor residual spraying (IRS) for malaria vector control. Visceral leishmaniasis was virtually eradicated at that time, possibly as a beneficial by-product of the MEP. However, with the discontinuation of MEP, VL re-emerged in the early 1980s [6,7]. DDT was later banned entirely by the Government of Bangladesh in 1998 as it was an environmental hazard [8].

In 2005, a memorandum of understanding (MoU) was signed among three countries (Bangladesh, India and Nepal) in the ISC, with the aim to eliminate VL as a public health problem across the region by 2015 [9]. This date was later extended to 2017 with the inclusion of Bhutan and Thailand in the consortium [10]. The target was to reduce the VL incidence to less than one case per 10,000 of the population annually at the upazila level in Bangladesh [9]. Between 1994 to 2013, the Directorate General of Health Services (DGHS) of Bangladesh reported 1,09,266 cases of VL and 329 VL related deaths from 37 endemic districts [7]; with about 50% of total VL cases being reported from five upazilas in the Mymensingh district. In the period after signing the MoU, extensive research activities were conducted to reduce VL transmission and provide treatment in coordination with the Special Programme for Research and Training in Tropical Diseases (TDR) at World Health Organization (WHO). This lead to a slow, but continued, reduction of VL cases until the elimination target was achieved and the first steps of the maintenance phase were initiated in 2017 [11].

Integrated vector management (IVM) is one of the most important elements in the regional elimination strategy [9]. However, for a significant period during the attack phase of the programme, from 1999 to early 2012, no VL vector control activities were performed in Bangladesh [7,12], and the number of cases increased every year during that time. A research programme in Bangladesh, India and Nepal showed that IRS using the synthetic pyrethroid (SP) deltamethrin 5WP was very effective against the VL vector if performed in accordance with guidelines [13,14]. Based on this, a pilot IRS trial was conducted in 2011 by the national programme using deltamethrin 5 WP, which proved to be successful. In 2012 a scale-up IRS operation was undertaken in eight highly endemic upazilas in four districts [7]. The Ministry of Health and Family Welfare, Government of Bangladesh organized several training courses for spraymen and supervisors. In parallel, WHO/TDR, along with programme managers and local researchers, developed a monitoring and evaluation toolkit for IRS [15]. This comprehensive toolkit should be used in order to provide an evidence base and explain possible reasons affecting the efficacy of IRS [16]. The present study will use this toolkit to monitor and document activities of IRS operations conducted by the national programme in Bangladesh using deltamethrin 5 WP.

## Methods

### Study sites and dates

**Fig 1:**
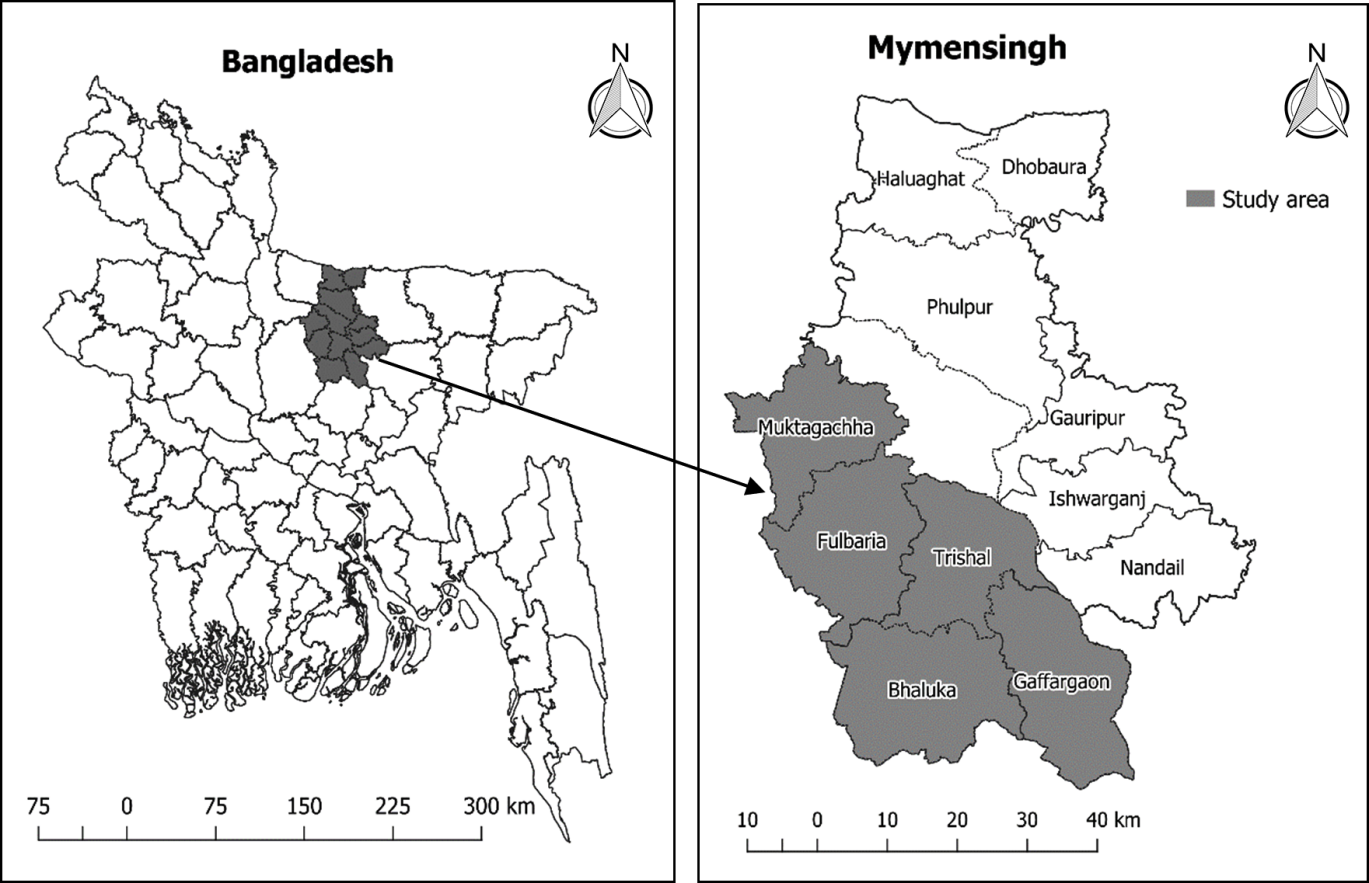
Study upazilas of Mymensingh district (note: the map was prepared using R rastering and programming).

In 2012, pre-monsoon (May-June) IRS was conducted in eight highly endemic upazilas, namely: Fulbaria, Trishal, Mukthagacha, Gaforgaon, Bhaluka of Mymensingh district (Fig 1), and Nagarpur, Madarganj, Terokhada upazilas of Tangail, Jamalpur and Khulna districts, respectively. The study was carried out from March to October of 2012 and included the following activities:

1. Observation of IRS activities using an observation check-list in all study upazilas.
2. Community satisfaction survey with a questionnaire: Household (HH) interviews in five study upazilas.
3. Vector density monitoring (pre- and post-IRS) using CDC light traps in one upazila in Fulbaria.
4. Bioassays on sprayed surfaces using the WHO cone method carried out in Fulbaria, Mukthagacha and Trishal upazilas.

### Sample size calculation

- **Observation of spraying squads:** The sample size for the number of squads to be observed was calculated based on the assumption that 80% (with 10% precision, 80% confidence level) of squads in each upazila had overall acceptable spraying performance. The minimum number of squads to be observed was 26 per upazila, and altogether 130 squads. To increase the precision we observed 136 squads (Fulbaria-30, Trishal-28, Bhaluka-26, Gaforgaon-26 and Mukthagacha-26).
- **Household acceptability survey:** The sample size for assessing people’s satisfaction with IRS was calculated assuming 85% of the houses in each upazila will accept IRS; with a precision of +/- 7% and a confidence interval of 95%; the required sample size was 100 HHs per upazila. In order to increase the strength of information we interviewed a total of 600 HHs (120 in each upazila).
- **Vector density measurement:** We calculated the sample size for sand fly density measurement based on the following assumptions: i) 50% sand fly reduction will be achieved by IRS, ii) ratio of the sample size considered the same in intervention and control areas, and iii) 90% power and alpha = 0.05. The minimum required sample size was determined to be 21 HHs in each treatment group: intervention (IRS) and control (no IRS). To increase the power of data we randomly selected 36 HHs from each treatment group for monitoring the sand fly densities.
- **Bioassay on sprayed surfaces:** The sample size calculation for bioassay experiment was calculated based on the following assumptions: i) average sand fly mortality per HH in intervention arm is about 80%, ii) average sand fly mortality per HH in control arm is about 20% (considering maximum allowable mortality for control cone) and iii) 80% power, 5% level of significance. Using a two-sample proportion test, the required number of HHs was 10 in each upazila for bioassay experiment where it required a total of 30 HHs. But we included 40 HHs (17, 12 and 11 HHs in Fulbaria, Trishal and Mukthagacha upazila, respectively) in IRS villages which may increase the power of this study. Similar number of HHs was included in the control (no IRS) villages. All HHs in both areas (IRS and control) were randomly selected.
- **Methods and forms:** Methods and forms used in the study can be found in the M&E toolkit (available at: http://www.who.int/tdr/publications/documents/irs_toolkit.pdf?ua=1)

### Description of the activities

#### Observation of spray team/squad

Trained research assistants were employed to observe and document the spraying activities of spraymen/spray squads using a standard checklist [15]. Two formed a team; each team observed 5 to 6 squads (a squad composed by 4 spraymen and 1 squad leader) per day.

#### Household acceptability survey

One month post-spraying, trained research assistants performed the HH IRS acceptability surveys using a structured questionnaire [15]. Each interviewed 20 to 25 HHs per day, usually the head of HH or, in his absence, his wife.

#### Vector density measurement in sprayed and unsprayed houses

Sand fly density was measured 2 weeks prior to IRS, at one and five months post-IRS in 36 sprayed and 36 unsprayed (control) HHs. Trained entomology technicians supervised by a research officer and one of the investigators performed the activities. In each HH, one CDC light trap was set up, in the corner of the main room (where the maximum number of members of the HH sleep at night), 2.5–5.0 cm from the wall and 15.0 cm from the floor, for two consecutive nights, from 18.00 hours to 06.00 hours [17]. The sand flies collected in each trap were transferred to test tubes using a mechanical aspirator and killed by adding chloroform-soaked cotton-wool to test tubes in the field. On return to the laboratory, sand flies were then separated by gender and identified up to species for the genus *Phlebotomus*, but not for other genera, using face contrast stereo binocular microscope to examine their external morphological characteristics [18,19]

#### Bioassay on sprayed (IRS HH) and unsprayed (control HH) surfaces

Bioassays were conducted in 80 HHs (40 IRS HHs and 40 control [unsprayed] HHs). In the night preceding the bioassay testing, sand flies were collected from unsprayed villages using manual aspirators and were kept (only female *P*. *argentipes* sand flies after separating them from other species) in a plastic pot and fed with sucrose solution in soaked cotton. In each house, 10–12 female *P*. *argentipes* were exposed for 30 minutes on each of the four inside walls of each HH, using the standard protocol for WHO plastic cones [20]. Tests were performed at the temperature of 27 ± 2°C and maintained 85% ± 10% relative humidity for 24 hours. Mortality of the exposed sand flies was recorded 24 hours post-exposure [21]. Corrected mortality was calculated, using the Abbot (1925) formula [22], as 100(Pi-C)/(100-C), where Pi and C are the percentage mortalities observed in the sprayed and unsprayed houses, respectively where control mortality should be within 5–20%. Tests were invalid in the case of control mortality exceeding 20% and those tests were repeated. The bioassays were performed at two post intervention time points for each test house: 1 and 4 months post-IRS.

### Statistical analysis

All field and laboratory data was checked, verified, cleaned of obvious errors and entered into databases using Microsoft Office Access 2007. The data analyses was performed using SPSS version 25 (SPSS Inc, Chicago, IL) and Stata version 12 (Stata Corp, College Station, TX). Descriptive statistics were used to explore the nature of the data. Frequency and percentages were used to analyze all the datasets (observation of spray team, household acceptability surveys, vector density measurement and bioassay). Due to the over-dispersed vector density measurements, the nonparametric Mann-Whitney U test was used to test whether there was a significance difference between intervention and control groups in between pre-intervention and post-intervention measurements. Wilcoxon Signed Rank test for paired sample was used to test whether there was any significance difference between baseline and follow-up. For each post-IRS timepoint, the percentage reduction in sand fly counts attributable to IRS was calculated as 100 [(mean post-intervention value for the intervention group-mean baseline value for the intervention group)-(mean post-intervention value for the control group-mean baseline value for the control group)]/(mean baseline value for the intervention group). To investigate the effect of vector density reduction we used the following formula:

$$Reduction\;rate\;(\%)=\frac{\left(\bar{I}-{\bar{I}}_{base})-(\bar{C}-{\bar{C}}_{base}\right)}{{\bar{I}}_{base}}\times 100$$

Where, ${\bar{I}}_{base}$ = average number of baseline sand fly in intervention group; ${\bar{C}}_{base}$ = average number of baseline sand fly in control group; $\bar{I}$ = average number of post intervention sand fly in intervention group; $\bar{C}$ = average number of post intervention sand fly in control group. The effect was negative and positive if the sand fly density decreased or increased post-intervention, respectively.

### Ethical approval

The study protocol (ID: BMRC/NREC/2010-2013/535) was approved by the ethical review committees of the Bangladesh Medical Research Council (BMRC), Dhaka, Bangladesh. On behalf of the national programme, Director Disease Control, DGHS had kindly given permission to observe spraying activities and interview spraying squads. Written informed consent was obtained from the head of each study household for active participation in the study.

## Results

### Training squad members (spraymen and squad leaders) had received and procedures

Each squad consists of five people: four spraymen and one squad leader. We observed IRS operations of 136 squads (544 spraymen and 136 squad leaders), in five endemic upazilas of Mymensingh district. Table 1 shows that 59.0%, 3.1% and 37.4% of the 544 spraymen received training for one, two and three days, respectively, before the IRS operation but all 136 squad leaders had training for three days. Only 14.0% of spraymen were involved in previous IRS activities. Regarding the procedures to approach houses for spraying, about 3.0% of squad leaders reported that there were only a few HHs who did not want to get their houses sprayed. Forty nine percent of squad leaders informed that they found some houses locked when they arrived. Almost all squads (98.5%) reported that they washed their spray pump at the end of the day and 72.8% of spraymen knew how to handle left-over insecticide or did not have any left-over (20.6%) [Table 1].

**Table 1:** Spraymen and squad leaders interview about indoor residual spraying in five upazilas of Mymensingh district [squad leader = 136, spraymen = 544 (%)]. All (136) squad leaders provided three days training. Values indicated in the table: without bracket = number of yes, within () = total participant, within [] = % of yes.

Statement	Five upazilas	Name of Upazila				
		Fulbaria	Gaforgaon	Mukthagacha	Trishal	Bhaluka
Spraymen who received training before IRS						
*Five upazilas*	540(544)[99.3]	120(120)[100]	100(104)[96.2]	104(104)[100]	112(112)[100]	104(104)[100]
*One day*	321(540)[59.0]	116(120)[96.7]	0(0)[0.0]	99(104)[95.2]	106(112)[94.6]	0(0)[0.0]
*Two days*	17(540)[3.1]	4(120)[3.3]	0(0)[0.0]	5(104)[4.8]	2(112)[1.8]	6(104)[5.8]
*Three days*	202(540)[37.4]	0(0)[0.0]	100(104)[96.2]	0(0)[0.0]	4(112)[3.6]	98(104)[94.2]
Spraymen ever involved in IRS activities	77(544)[14.2]	33(120)[27.5]	4(104)[3.8]	8(104)[7.7]	24(112)[21.4]	8(104)[7.7]
Squad leaders found households refuse to have IRS	4(544)[2.9]	0(0)[0.0]	0(0)[0.0]	2(26)[7.7]	1(28)[3.6]	1(26)[3.8]
Squad leaders found houses locked during spraying	67(544)[49.3]	20(30)[66.7]	0(0)[0.0]	22(26)[84.6]	22(28)[78.6]	3(26)[11.5]
Spraymen (spraying squad) washed pumps at the end of every day	134(544)[98.5]	29(30)[96.7]	26(26)[100]	26(26)[100]	27(28)[96.4]	26(26)[100]
What do you do with leftover insecticides?						
*Correct*	99(136)[72.8]	18(30)[60.0]	14(26)[53.8]	21(26)[80.8]	22(28)[78.6]	24(26)[92.3]
*Incorrect*	9(136)[6.6]	1(30)[3.3]	1(26)[3.8]	3(26)[11.5]	2(28)[7.1]	2(26)[7.7]
*Does not have leftover*	28(136)[20.6]	11(30)[36.7]	11(26)[42.3]	2(26)[7.7]	4(28)[14.3]	0(26)[0.0]

### Observation of IRS operation

#### General preparation

IRS activities of 136 squads were observed in five upazilas [Table 2]. Squad leaders were present during spraying for only 64.0% of the houses sprayed. Only one upazila, Mukthagacha, had squad leaders present during spraying for 100% of the houses [Table 2]. Whilst spraying, 43.4% of spraymen correctly filled their pumps, but in one upazila—Gaforgaon—only 7.7% had correctly filled their pumps. Table 2 shows that 41.8% of squads had properly mixed (100 gm sachet mix with 8 liters of water) deltamethrin prior to spraying, but in Gaforgaon it was only 7.7%. About seventy three percent (72.8%) of spraying squads informed HH members to prepare their houses prior to spraying according to the toolkit [Table 2]. Preparation includes taking out or covering food items, utensils, cloths, and others. Domestic animals should also be kept outside during spraying. 79.4% of squad members asked HHs members to stay outside during and after spraying. Very few (2.2%) squads knew all the parameters for marking sprayed house other than writing down the squad number (83.8%).

**Table 2:** Observation of national indoor residual spraying activities in five upazilas of Mymensingh district. Values indicated in the table: without bracket = number of yes, within () = total participant, within [] = % of yes.

Statement	Five upazilas	Fulbaria	Gaforgaon	Mukthagacha	Trishal	Bhaluka
Supervisor was present during spraying	87(136)[64.0]	13(30)[43.33]	15(26)[57.7]	26(26)[100]	12(28)[42.9]	21(26)[80.8]
Correct filling of the pump by squads members	59(136)[43.4]	17(30)[56.7]	2(26)[7.7]	14(26)[53.8]	17(28)[60.7]	9(26)[34.6]
Proper mixing of insecticides	56(136)[41.8]	17(30)[56.7]	2(26)[7.7]	14(26)[53.8]	16(26)[57.1]	7(26)[26.9]
Instruction given to households						
Stay outside while spraying and re-entering after spraying	108(136)[79.4]	25(30)[83.3]	24(26)[92.3]	19(26)[73.1]	15(28)[53.6]	25(26)[96.2]
Prepare house for spraying	99(136)[72.8]	25(30)[83.3]	18(26)[69.2]	16(26)[61.5]	15(28)[53.6]	25(26)[96.2]
Use of safety measures						
*Mask*	363(544)[66.7]	102(120)[85.0]	48(104)[46.2]	81(104)[77.9]	53(112)[47.3]	79(104)[76.0]
*Goggles*	196(544)[36.0]	79(120)65.8]	19(104)[18.3]	36(104)[34.6]	35(112)[31.3]	27(104)[(26.0]
*Glove*	122(544)[22.6]	56(120)[46.7]	7(104)[6.7]	34(104)[32.7]	7(112)[6.3]	18(104)[17.3]
*Coat/ Apron*	483(544)[88.7]	112(120)[93.3]	78(104)[75.0]	97(104)[93.3]	99(112)[88.4]	97(104)[93.3]
*Cap*	275(544)[50.5]	33(120)[27.5]	49(104)[47.1]	92(104)[88.5]	42(112)[37.5]	59(104)[56.7]
*Boot*	0(544)[0.0]	0(120)[0.0]	0(104)[0.0]	0(104)[0.0]	0(112)[0.0]	0(104)[0.0]
*Towel*	467(544)[85.8]	100(120)[83.3]	79(104)[76.0]	104(104)[100]	100(112)[89.3]	84(104)[80.8]
*Soap*	429(544)[78.9]	96(120)[80.0]	66(104)[63.5]	104(104)[100]	91(112)[81.3]	72(104)[69.2]
Proper spraying						
*Spraying bottom to top*	375(544)[69.6]	95(120)[79.2]	92(104)[88.5]	48(104)[46.2]	58(112)[51.8]	86(104)[82.7]
*Clockwise spraying inside the room*	191(544)[35.1]	57(120)[47.5]	40(104)[38.5]	13(104)[12.5]	45(112)[40.2]	36(104)[34.6]
*Vertical swath (75 cm)*	288(544)[52.9]	76(120)[63.3]	53(104)[51.0]	42(104)[40.4]	65(112)[58.0]	52(104)[50.0]
*Swath overlap (5 cm)*	186(544)[34.2]	58(120)[48.3]	12(104)[11.5]	30(104)[28.8]	38(112)[33.9]	48(104)[46.2]
*Proper distance of nozzle from the surface (45 cm)*	250(544)[46.0]	66(120)[55.0]	53(104)[51.0]	44(104)[42.3]	41(112)[36.6]	46(104)[44.2]
*Correct discharge rate by pump (790 ml / min)*	216(544)[39.7]	84(120)[70.0]	7(104)[6.7]	56(104)[53.8]	65(112)[58.0]	4(104)[3.8]
Marking of sprayed houses						
*HH ID*	26(136)[19.1]	19(120)[63.3]	1(104)[3.8]	0(104)[0.0]	1(112)(3.6)	5(104)[19.2]
*Date of spray*	40(136)[29.4]	0(120)[0.0]	23(104)[88.5]	0(104)[0.0]	11(112)[39.3]	6(104)[23.1]
*Spray cycle*	17(136)[12.5]	0(120)[0.0]	0(104)[0.0]	5(104)[19.2]	12(112)[42.9]	0(104)[0.0]
*Team number*	114(136)[83.8]	30(120)[100]	11(104)[42.3]	26(104)[100]	28(112)[100]	19(104)[73.1]
*Available room and number of room sprayed*	3(136)[2.2]	0(120)[0.0]	1(104)[3.8]	0(104)[0.0]	1(112)[3.6]	1(104)[3.8]
Number of rooms in each house sprayed						
*Satisfactory*	15(136)[11.0]	9(120)[30.0]	1(104)[3.8]	1(104)[3.9]	0(112)[0.0]	4(104)[15.4]
*Partial*	115(136)[84.6]	18(120)[60.0]	25(104)[96.2]	22(104)[84.6]	28(112)[100.0]	22(104)[84.6]
*Not at all*	6(136)[4.4]	3(120)[10.0]	0(104)[0.0]	3(104)[11.5]	0(112)[0.0]	0(104)[0.0]

#### Use of safety measures

Regarding the use of personal protection equipment while spraying, only 66.7% of spraymen used a mask across all the upazilas, but use was higher in Fulbaria (85%). The use of goggles was even lower in all upazilas (36.0%), and gloves even less so (22.6%) except in Fulbaria (46.7%). However, the use of a coat/apron was more frequent (88.7%). In all sites, 50.5% of spraymen wore caps, but this was higher in Mukthagacha (88.5%) although much less in Fulbaria (27.5%). No spraymen used boots during our observations. However, towels (85.8%) and soap (78.9%) were frequently used at the end of the working day [Table-2].

#### Spraying technique

In all sites, the proper spraying technique from bottom to top was practiced on average by 69.6% of the sprayers. Clockwise spraying inside the room was practiced by 35.1% of sprayers. Only 52.9% of spraymen properly maintained vertical swath (75cm) and only 34.2% of spraymen maintained swath overlaps (5cm). Maintaining the requisite distance of the nozzle from the spraying surface (45cm) was observed in 46.0% of the spraying activities and the correct discharge rate by pump (790ml/minute) was done correctly in 39.7% of the observations. It was seen that 84.6% of spraying squads performed partial spraying in the rooms, while only 11.0% of squads performed complete IRS [Table-2].

### Community satisfaction with IRS

All the respondents in the 600 HHs of the five study upazilas mentioned that their houses had been sprayed with insecticide [Table-3] and 94% said that their living rooms and cattle sheds had also been sprayed. Only 36.2% of interviewees mentioned that they had been informed about IRS in advance. The majority of respondents (85.3%) said that they were happy with the IRS activities. Of the 15% respondents who were unhappy, the main source of complaint (76% of this subgroup) was that they did not recognize that the spraying killed any insects. Although the majority of the respondents (81.7%) received advice to remove or cover clothes, food/utensils, children and animals prior to spraying, only 13.3% reported that they were instructed not to enter the house after spraying was completed. Only 7.2% of respondents had been informed about possible side effects (itching, burning of skin, dizziness, cough, etc.) of the spraying and 91.3% reported that they were not advised to abstain from re-plastering (mud wall) or re-painting (pacca/tin house) their houses after spraying [Table 3].

**Table 3:** Household acceptability survey on indoor residual spraying in five upazilas of Mymensingh district. Values indicated in the table: without bracket = number of yes, within () = total participant, within [] = % of yes.

Statement		Five upazilas	Fulbaria	Gaforgaon	Mukthagacha	Trishal	Bhaluka
Did your house sprayed with insecticide?		600(600)[100]	120(120)[100]	120(120)[100]	120(120)[100]	120(120)[100]	120(120)[100]
Do they sprayed in your living room and cattle shed?		569(600)[94.8]	115(120)[95.8]	111(120)[92.5]	117(120)[97.5]	117(120)[97.5]	109(120)[90.8]
If no, how many rooms were not sprayed?	1 room	22(600)[3.7]	5(120)[4.2]	8(120)[6.7]	2(120)[1.7]	2(120[1.7]	5(120)[4.2]
	2 rooms	6(600)[1.0]	0(120)[0.0]	1(120)[0.8]	0(120)[0.0]	1(120)[0.8]	4(120)[3.3]
	3 rooms	1(600)[0.2]	0(120)[0.0]	0(120)[0.0]	1(120)[0.8]	0(120)[0.0]	0(120)[0.0]
	4 rooms	2(600)[0.3]	0(120)[0.0]	0(120)[0.0]	0(120)[0.0]	0(120)[0.0]	2(120)[1.7]
Have you been informed before spraying?		217(600)[36.2]	63(120)[52.5]	19(120)[16.0]	63(120)[52.5]	36(120)[30.0]	36(120)[30.0]
Are you satisfied with spraying?		509(600)[85.3]	95(120)[79.2]	109(120)[91.6]	102(120)[85.0]	93(120)[77.5]	110(120)[93.2]
If no, the reasons:	No meaning for this insecticide spraying	1(600)[2.0]	1(120)[4.5]	0(120)[0.0]	0(120)[0.0]	0(120)[0.0]	0(120)[0.0]
	Rude behavior of spraymen	1(600)[2.0]	1(120)[4.5]	0(120)[0.0]	0(120)[0.0]	0(120)[0.0]	0(120)[0.0]
	Spraymen sprayed like water (diluted spray)	12(600)[24.0]	5(120)[22.7]	0(120)[0.0]	2(120)[11.1]	5(120)[62.5]	0(120)[0.0]
	After spraying did not kill the insects	38(600)[76.0]	17(120)[77.3]	1(120)[50.0]	16(120)[88.9]	4(120)[50.0]	0(120)[0.0]
	Insecticide has side effects	1(600)[2.0]	0(120)[0.0]	1(120)[50.0]	0(120)[0.0]	0(120)[0.0]	0(120)[0.0]
	Bed smell/odor	0(600)[0.0]	0(120)[0.0]	0(120)[0.0]	0(120)[0.0]	0(120)[0.0]	0(120)[0.0]
Before spraying did you get any advice like removing or covering cloths, food/utensils; and children, animals take out from house and cattle shed respectively?		490(600)[81.7]	95(120)[79.2]	87(120)[72.5]	110(120)[91.7]	88(120)[73.3]	110(120)[91.7]
Have you been advised about the time you should wait to enter the house after spraying is completed?		80(600)[13.3]	5(120)[4.2]	7(120)[5.8]	29(120)[24.2]	12(120)[10.0]	27(120)[22.5]
If yes, mention time (in minutes)	≤30	24(600)[4.0]	2(120)[1.7]	4(120)[3.3]	7(120)[5.8]	4(120)[3.3]	7(120)[5.8]
	60	42(600)[7.0]	3(120)[2.5]	3(120)[2.5]	18(120)[15.0]	4(120)[3.3]	14(120)[16.7]
	120	14(600)[2.3]			4(120)[3.3]	4(120)[3.3]	6(120)[5.0]
After spraying do you have any side effect? (multiple answer can be)		43(600)[7.2]	3(120)[2.5]	24(120)[20.0]	0(120)[0.0]	2(120)[1.7]	12(120)[10.0]
If yes, what effects	Cough	4(600)[0.7]	0(120)[0.0]	0(120)[0.0]	0(0.0)	0(120)[0.0)	2(120)[0.3]
	Dizziness	4(600)[0.7]	0(120)[0.0]	0(120)[0.0]	0(0.0)	0(120)[0.0]	4(120)[0.7]
	Fever	10(600)[1.7]	1(120)[0.8]	9(120)[7.5]	0(0.0)	0(120)[0.0]	0(120)[0.0]
	Headache	2(600)[0.3]	0(120)[0.0]	2(120)[1.7]	0(0.0)	0(120)[0.0]	0(120)[0.0]
	Itching	9(600)[1.5]	0(120)[0.0]	8(120)[6.7]	0(0.0)	0(120)[0.0]	1(120)[0.2]
	Running nose	5(600)[0.8]	1(120)[0.8]	1(120)[0.2]	0(0.0)	1(120)[0.2]	2(120)[0.3]
	Conjunctivitis	0(600)[0.0]	0(120)[0.0]	0(120)[0.0]	0(0.0)	0(120)[0.0]	0(120)[0.0]
	Burning of skin	6(600)[1.0]	1(120)[0.8]	2(120)[0.3]	0(0.0)	1(120)[0.2]	3(120)[2.5]
	Insomnia	0(600)[0.0]	0(120)[0.0]	0(120)[0.0]	0(0.0)	0(120)[0.0]	0(120)[0.0]
	Sneezing	0(600)[0.0]	0(120)[0.0]	0(120)[0.0]	0(0.0)	0(120)[0.0]	0(120)[0.0]
	Stomach ache	1(600)[0.2]	0(120)[0.0]	1(120)[0.2]	0(0.0)	0(120)[0.0]	0(120)[0.0]
	Others	2(600)[0.3]	0(120)[0.0]	1(120)[0.2]	0(0.0)	0(120)[(0.0]	0(120)[0.0]
How long are you advised not to mud plaster or lime plaster or paint the wall after spraying?							
Not advised		548(600)[91.3]	104(120)[86.7]	117(120)[97.5]	103(120)[85.8]	110(120)[91.7]	114(120)[95.0]
Adivsed (in days)	7	5(600)[0.8]	1(120)[0.8]	1(120)[0.8]	3(120)[2.5]	0(120)[0.0]	0(120)[0.0]
	30	7(600)[1.2]	2(120)[1.7]	0(120)[0.0]	2(120)[1.7]	2(120)[1.7]	1(120)[0.8]
	90	15(600)[2.5]	7(120)[5.8]	1(120)[0.8]	1(120)[0.8]	3(120)[2.5]	3(120)[2.5]
	150	7(600)[1.2]	0(120)[0.0]	1(120)[0.8]	4(120)[3.3]	0(120)[0.0]	2(120)[1.7]
	180	18(600)[3.0]	6(120)[5.0]	0(120)[0.0]	7(120)[5.8]	5(120)[4.2]	0(120)[0.0]

### Effect of IRS on sand flies

Table 4 shows the absolute numbers, species, and physiological status of sand flies collected in the households in both treatment groups (IRS and control) of the study. A total of 4132 sand flies were trapped in three time intervals (at baseline, 1 month and 5 months post-intervention), of which 3310 (80.1%) were *P*. *argentipes* and 822 other species [Table 4]. Of all *P*. *argentipes*, 46.5% (1540) were males and 53.5% (1770) females; including 20.1% (666) gravid females and only 2 blood-fed females.

**Table 4:** Sand flies collected in all study households by species, sex, gravidity, feeding status, and episode of collection, Fulbaria, Mymensingh, Bangladesh in 2012.

Species	Gender	Status	IRS and control household				IRS household				Control household			
			Collection interval [%]				Collection interval [%]				Collection interval [%]			
			Baseline	1-month post-intervention	5-months post-intervention	Total	Baseline	1-month post-intervention	5-months post-intervention	Total	Baseline	1-month post-intervention	5-months post-intervention	Total
*Phlebotomus argentipes*														
	Female		526 [55.90]	605 [51.89]	639 [53.12]	1770 [53.47]	377 [60.13]	354 [45.68]	384 [51.00]	1115 [51.74]	149 [47.45]	251 [64.19]	255 [56.67]	655 [56.71]
		Gravid	201 [21.36]	252 [21.61]	213 [17.71]	666 [20.12]	158 [25.20]	155 [20.00]	141 [18.73]	454 [21.07]	43 [13.69]	97 [24.81]	72 [16.00]	212 [18.35]
		Fed	0 [0]	0 [0]	2 [0.17]	2 [0.06]	0 [0]	0 [0]	0 [0]	0 [0]	0 [0]	0 [0]	2 [0.44]	2 [0.17]
		Neither	325 [34.54]	353 [30.27]	394 [32.75]	1072 [32.39]	219 [34.92]	199 [25.68]	243 [32.27]	661 [30.67]	106 [33.76]	154 [39.39]	151 [33.56]	411 [35.58]
	Male		415 [44.10]	561 [48.11]	564 [46.88]	1540 [46.53]	250 [39.87]	421 [54.32]	369 [49.00]	1040 [48.26]	165 [52.55]	140 [35.81]	195 [43.33]	500 [43.29]
	Total		941 [67.70]	1166 [78.41]	1203 [95.86]	3310 [80.11]	627 [67.42]	775 [75.91]	753 [98.95]	2155 [79.46]	314 [68.26]	391 [83.91]	450 [91.09]	1155 [81.34]
Other species			449	321	52	822	303	246	8	557	146	75	44	265
**Total**			**1390**	**1487**	**1255**	**4132**	**930**	**1021**	**761**	**2712**	**460**	**466**	**494**	**1420**

The average numbers of *P*. *argentipes* per household per night per trap in IRS and control arms were 4.36 and 8.31 for both (combined) male and female, 2.29 and 3.47 for only male, 2.71 and 5.12 for only female, 0.60 and 2.19 for gravid female respectively [Table 5]. At baseline (pre-intervention), the number of *P*. *argentipes* was significantly lower in the IRS treatment group compared with the control group in both male and female (p < 0.05), only female (p<0.05), and gravid female (p<0.01) whereas no significance difference was observed in only male (p = 0.177). Similarly, at 1 month post-IRS, there was a significant difference in *P*. *argentipes* densities between the treatment groups for both male and female (p<0.05) and for only male (p<0.05). But no significant difference was found for only female (p = 0.318) and gravid female (p = 0.106). At 5 months post-IRS, no significant difference in *P*. *argentipes* densities was observed between treatment groups for both male and female (p = 0.282), for only male (p = 0.350), for only female (p = 0.261) and for gravid female (p = 0.264). This indicates that IRS is able to control the increase of only male *P*. *argentipes* density in the IRS areas up to one month post spraying (at first follow-up). But it fails to control the *P*. *argentipes* density in IRS houses at five months (second follow-up). Table 5 shows that one month after IRS the *P*. *agrentipes* density was reduced by 22.61% and 118.79% in both (combined) male and female, and only male which dropped to 6.37% and 53.94% at five months respectively. However, no reduction was found in only female and gravid female *P*. *argentipes* density.

**Table 5:** *Phlebotomus argentipes* sand fly density per house (trap) per night in IRS and control treatment groups for both male and female, only male, only female and graved female.

Time/sand fly density (count)	IRS, Mean (95% CI)	Control, Mean (95% CI)	p-value	% of reduction by intervention
**Both Male and Female *P*. *argentipes***				
***At Baseline***				
Mean ± SD	4.36 ± 5.73	8.71 ± 11.37	0.015*	
(95% CI)	(2.42–6.30)	(4.86–12.55)		
***At first month follow-up***				
Mean ± SD	5.43 ± 6.11	10.76 ± 7.50	0.023*	-22.61%
(95% CI)	(3.36–7.50)	(7.27–14.26)		
Mean difference from base line ± SD	1.07 ± 6.11	2.05 ± 10.32	0.521	
(95% CI)	(-1.0–3.14)	(-1.44–5.55)		
p-value	0.310	0.243		
***At 5***^***th***^ ***months follow-up***				
Mean ± SD	5.83 ± 5.79	10.46 ± 7.79	0.282	-6.37%
(95% CI)	(3.87–7.79)	(6.24–14.67)		
Mean difference from base line ± SD	1.47 ± 05.79	1.75 ± 12.46	0.093	
(95% CI)	(-2.47–5.96)	(-0.49–3.43)		
p-value	0.310	0.310		
**Only Male *P*. *argentipes***				
***At Baseline***				
Mean ± SD	2.29 ± 3.24	3.47 ± 4.61	0.177	
(95% CI)	(1.20–3.39)	(1.91–5.03)		
***At first month follow-up***				
Mean ± SD	1.94 ± 3.08	5.85 ± 5.83	0.001**	-118.79%
(95% CI)	(0.90–2.99)	(3.88–7.82)		
Mean difference from base line ± SD	-0.35 ± 6.11	2.38 ± 5.83	0.183	
(95% CI)	(-1.39–0.70)	(0.41–4.35)		
p-value	0.265	0.232		
***At 5***^***th***^ ***months follow-up***				
Mean ± SD	2.71 ± 2.77	5.12 ± 6.49	0.350	-53.94%
(95% CI)	(1.77–3.64)	(1.66–7.32)		
p-value	0.719	0.608		
**Only Female *P*. *argentipes***				
***At Baseline***				
Mean ± SD	2.07 ± 2.91	5.24 ± 7.35	0.001**	
(95% CI)	(1.08–3.06)	(2.75–7.72)		
***At first month follow-up***				
Mean ± SD	3.49 ± 3.71	4.92 ± 4.83	0.318	83.89%
(95% CI)	(2.23–4.74)	(3.28–6.55)		
Mean difference from baseline ± SD	1.42 ± 3.71	-0.32 ± 4.83	0.006**	
(95% CI)	(0.16–2.67)	(-1.96–1.31)		
p-value	0.031*	0.999		
***At 5***^***th***^ ***months follow-up***				
Mean ± SD	3.13 ± 3.44	5.33 ± 6.27	0.261	66.44%
(95% CI)	(1.96–4.29)	(3.21–7.45)		
Mean difference from baseline ± SD	0.42 ± 2.77	0.09 ± 6.27	0.007**	
(95% CI)	(-0.52–1.35)	(-2.03–2.21)		
p-value	0.164	0.377		
**Only gravid female *P*. *argentipes***				
***At Baseline***				
Mean ± SD	0.60 ± 0.85	2.19 ± 2.13	<0.001**	
(95% CI)	(0.31–0.89)	(1.47–2.91)		
***At first month follow-up***				
Mean ± SD	1.35 ± 1.68	2.15 ± 2.16	0.106	132.56%
(95% CI)	(0.78–1.92)	(1.44–2.88)		
Mean difference from base line ± SD	0.75 ± 1.68	-0.04 ± 2.16	0.002**	
(95% CI)	(0.18–1.32)	(-0.77–0.69)		
p-value	0.005**	0.914		
***At 5***^***th***^ ***months follow-up***				
Mean ± SD	1.00 ± 1.11	1.96 ± 2.71	0.264	106.98%
(95% CI)	(0.62–1.38)	(1.04–2.87)		
Mean difference from base line ± SD	0.40 ± 1.11	-0.23 ± 2.71	0.001**	
(95% CI)	(0.02–0.78)	(-1.15–0.68)		
p-value	0.063	0.771		

Significance at the 5% and 1% error level is indicated by * and **, respectively

**Fig 2:**
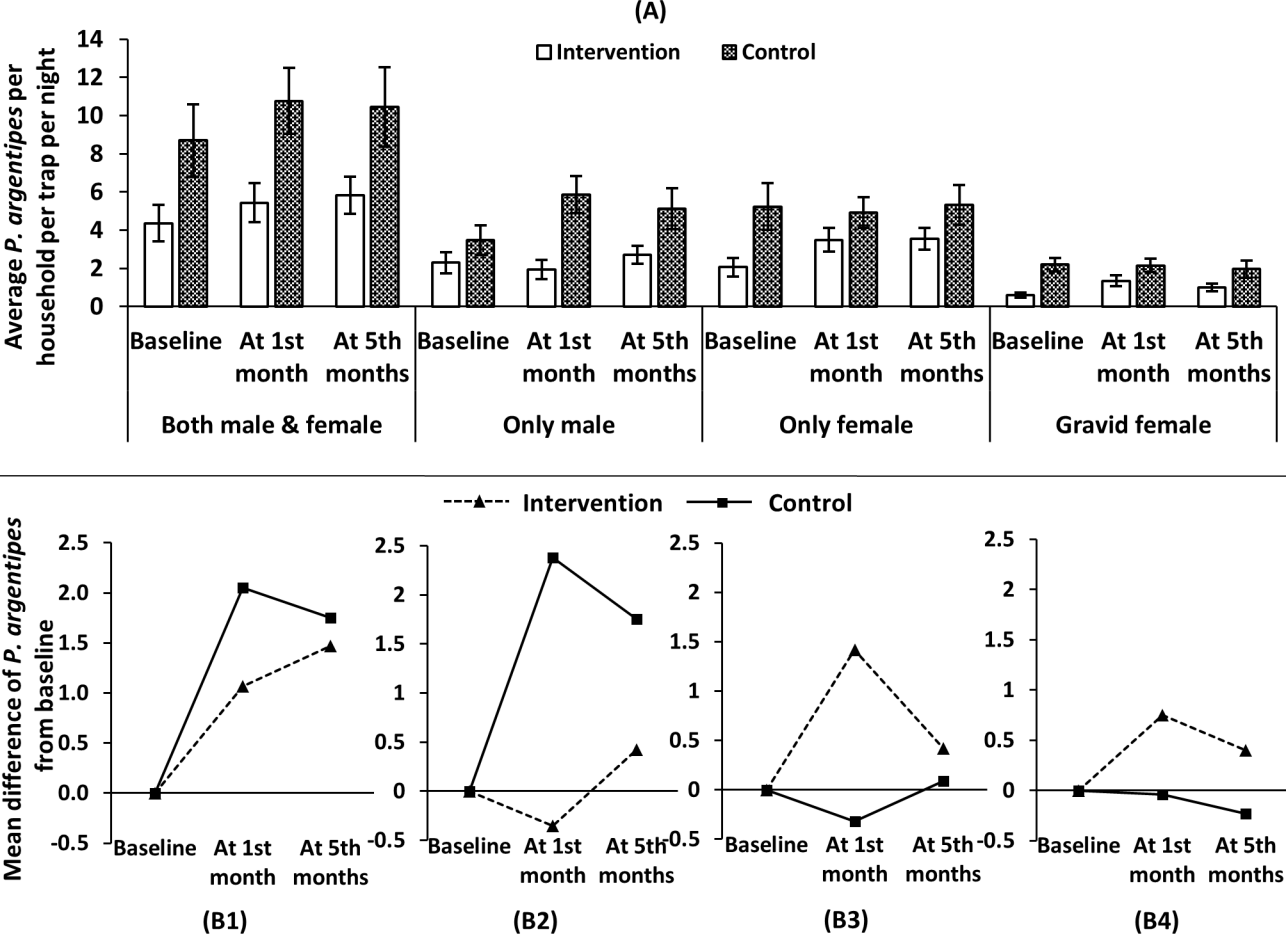
Average number of *Phlebotomus argentipes* per household per night per trap for both (combined) male and female, only male, only female and gravid female (A) and mean difference of *P*. *argentipes* sand flies compared to baseline at two measurement points for both (combined) male and female, only male, only female and gravid female (B1-4) in Fulbaria, Mymensingh district.

Fig 2(A) shows that the average number of *P*. *argentipes* sand flies per household per night per trap increase in both IRS and control treatment groups after one month post-IRS but the increase was more pronounced in the control group for both (combined) male and female. Similar increases happened in female and gravid female *P*. *argentipes* sand flies as well but the increase was not pronounced in both IRS and control treatment groups. But reduction of density was observed only in male *P*. *argentipes* sand flies in IRS treatment group and was increased in the control treatment group [Fig 2(A)]. There is no significant difference between the treatment groups compared with their baseline measurements apart from male *P*. *argentipes* sand flies. At 5-months post-intervention, there was no significant difference between the two treatment groups from baseline for all categories of *P*. *argentipes* sand flies [Fig 2(B)]. On an average, one *P*. *argentipes* sand flies increased in the sprayed areas per HH per night per trap compared to baseline at first follow-up which is two in control areas for both (combined) male and female. In sprayed areas, only male *P*. *argentipes* sand flies density reduced on an average 0.5 compared to baseline, which was 2.5 increases in the control areas at first month of IRS whereas only female increase was 1.5 and about one increase for gravid female [Fig 2(B)].

### WHO cone bioassays on sprayed surfaces

**Fig 3:**
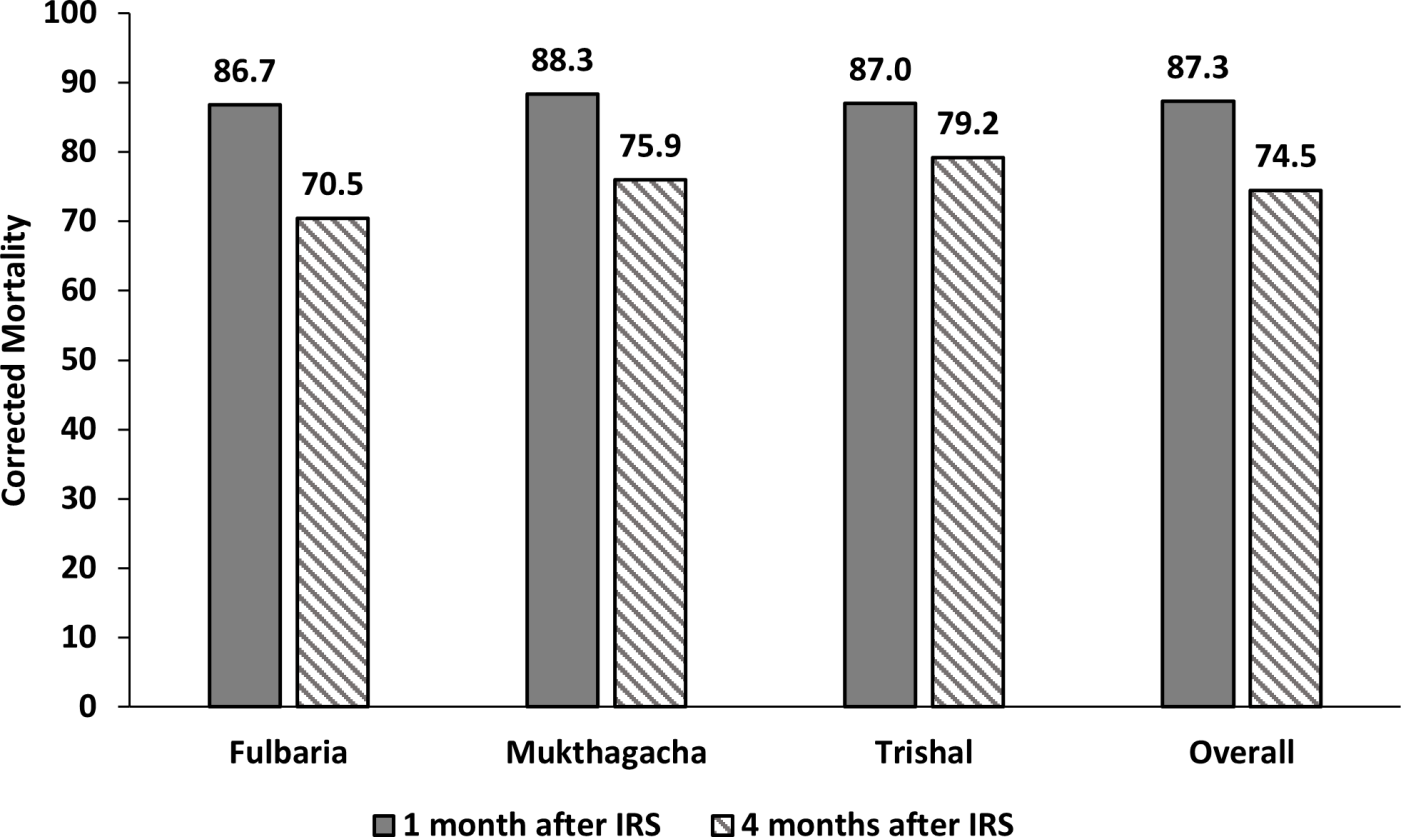
WHO cone bioassays on sprayed surfaces in three upazilas of Mymensingh district. Sand fly mortality after 24 hours corrected using Abbot formula.

Bioassays was conducted in three upazilas, namely: Fulbaria, Mukthagacha and Trishal. The mean corrected mortality of female *P*. *argentipes* sand flies was 87.3% (CI = 82.3%–92.3%) and 74.5% (CI = 69.3%–79.7%) at one and four months after spraying [Fig 3] showing that the insecticidal effect rapidly disappeared.

## Discussion

Spraymen and squad leaders are hired temporarily according to the requirements of the sub-district and in accordance with the micro-action plan of IRS. Spraymen training is compulsory prior to the IRS operation. The present study identified that only 37.5% of spraymen had received a full training in accordance with the national guidelines. As a result, the overall performance of house spraying was poor. Issues with the late release of fund, low daily wage of spraymen and squad leaders, and local pressure are hindering the timely recruitment of spraymen and squad leaders. Due to this, the national programme is facing challenges when training the spraymen. As no IRS was conducted for VL vector control from 1998 to early 2012 in the country [7,12], when DDT was banned [8], more than 85% of spraymen were never involved in IRS activities before. Though the acceptance of IRS activities was found to be very high (97%) in the study areas, more than 50% spraying squads found that there was no one home while they were in the village. This points to a communication gap between the community and the service provider. Often, the quality of IRS could not be ensured by proper supervision: the present study established that less than 44% of supervisors were present in Fulbaria and Trishal upazilas during the spraying activities [7]. Over- or under-concentration of the insecticide solution depends on the correct filling of the pumps; proper mixing of the insecticide and correct spraying technique ensures the uniform distribution of insecticides on sprayed surfaces. We found these two indicators to be substandard in all upazilas and extremely poor in two particular upazilas. Personal protective equipment is key to shield a person from any occupational hazard. However, as these were not distributed, we observed that none of the squad members were wearing boots. The use of goggles and gloves were also found to be low in all sites due to weak supervision. Though adequate training and proper supervision are essential for ensuring proper spraying, we observed substandard spraying techniques (ranges from 34.2%– 52.9% correct technique) except for spraying from bottom to top. Marking of sprayed houses is important for their supervision and identification; however, we found poor compliance with the guidelines, apart from mentioning the team number (about 84%).

Despite the many limitations, the communities positively accepted the IRS operations as they had not experienced any IRS or other vector control activities for a long time [7,12]. Understandably, people in the endemic communities do not want to suffer from insect nuisance, particularly VL, so they welcomed IRS activities because ‘something is better than nothing’. A previous study described how community members suffer from loss of earnings and how the symptoms of VL affect their wellbeing; therefore poor householders resorted to desperate actions, like the sale or rent of their assets, taking out loans, etc. to cover the expenditures of diagnostics and treatment [23]. The national programme should ensure that high quality IRS is delivered to get the maximal results. Substandard application of insecticides is also a potential cause of insecticide resistance since sub-lethal doses or poor quality insecticides enhance resistance development. Several studies have reported DDT resistance in *P*. *argentipes* [16,24,25] and it is essential that synthetic pyrethroids remain effective.

IRS is highly effective in quickly reducing vector densities underlining the need to continue the operations in all three countries of the ISC even during the consolidation and maintenance phases of VL elimination. Based on our study results the following recommendations can be made:

- **Community sensitization:** The community must be properly sensitized before organizing an IRS operation so that people are present when the spraying squad arrives.
- **IRS coverage:** High coverage along with proper spraying is crucial for getting the community effect. IRS was found very effective in the above mentioned TDR/WHO study [13,14,26] when investigated by a researcher in a controlled situation. However, it was found to be substandard when conducted by the national programme in India and Nepal [27]. 36.0% supervisors were absent from the squads during our study but proper supervision and monitoring are essential for high quality IRS.
- **Proper training:** Proper staff training is crucial not only for conducting high quality IRS but also essential to prevent spraymen from insecticidal hazards. In the present study we noticed that spraymen were not cautious about personal safety. They were not properly trained on how to handle insecticides safely and had not received essential personal protection equipment; similar observations were made in India and Nepal [27].
- **Quality of IRS:** The quality of the overall spraying has to be ensured. It was not satisfactory in all upazilas, and similar findings have been found in India and Nepal as well [27]. Correct IRS should be ensured through proper supervision and monitoring. The M&E tools for IRS [15] developed for the SEA region [28] should be used by the national programme.
- **Insecticide concentration:** Accurate insecticide concentration should be maintained during spraying to ensure the long lasting effects of IRS. A study in Bangladesh, India and Nepal has shown the low concentration of insecticides on the wall after IRS [28] due to poor spraying technique. A later study in India has also found that the DDT concentration on the sprayed walls was insufficient (i.e. below 80% of target dose of 1 g ai/m^2^ [16]. This may be the main reason why no significant vector reduction was detected except for male *P*. *argentipes* at one month after IRS in our study.

IRS is a huge operation where each step needs to include training of human resources as well as proper management. The experienced people involved in the malaria eradication programme carried out in the 1970s had all retired prior to the VL elimination programme so the national programme faced challenges to prepare a new cohort of spraymen and supervisors to handle such a complex operation.

In Bangladesh, the Director of the Central Medical Store Deport (CMSD), DGHS under the Ministry of Health and Family Welfare procured the insecticide and distributed the required quantity to the local hospitals (Upazila Health Complexes, UHCs). The Director of Disease Control, DGHS is responsible for conducting the national IRS operation through district managers (Civil Surgeon) and sub-districts managers. Filed operations of IRS in the respective UHC are managed by Upazila Health & Family Planning Officer (UH&FPO).

The responsibility for supervision and monitoring of IRS is to be done by the following officers:

○ Visits by Director of Disease Control, along with his/her team all UHCs
○ Visits by officials from WHO Country Office to some places
○ Visits by Deputy Director, Disease Control or Deputy Programme Manager (Kala-azar) to some areas
○ Civil Surgeon/Medical Officer-Disease Control/Health Superintended from respective districts may visit some UHCs
○ Senior Entomologist/District Entomologists are assigned to each UHC for full time supervision and monitoring of the IRS operation
○ UH&FPO (Upazila Health & Family Planning Officer)/Medical Officer-Kala-azar/Health Inspector from each UHC is responsible for supervising and monitoring daily IRS operation
○ External expert is occasionally assigned by the national programme (Director, CDC, DGHS) for external quality control

Integrated vector management is one of the most important elements mentioned in the regional VL elimination strategy. IRS is an effective vector control tool but it is an expensive operation. Therefore, it is recommended that the national programme ensure: (1) procurement of quality insecticide, (2) proper training of human resources involved in IRS operation, (3) proper monitoring and supervision during spraying, (4) regular vector surveillance and bioassays on sprayed surfaces, (5) routine testing of vector susceptibility and (6) community sensitization.

Since the launch of the VL elimination programme in 2005, this is the first study conducted in Bangladesh where national IRS operation was monitored by a third party (researcher) and substantial information was generated out of it. Bangladesh, along with other countries in the region, are still heavily relying on IRS so we strongly feel this information can guide national programmes for conducting well coordinated IRS operations. A limitation of the study includes the condition that we had to comply with the micro action plan made by the sub-district managers (UH&FPO) for all monitoring visits as we monitored their IRS activities. In such a case, there could have been a chance of leakage about the monitoring visits to IRS squads from beforehand. To avoid such circumstances we did not disclose our detailed plan of visits to local health authorities so that the quality and validity of data were ensured.

In conclusion, the national programme should routinely apply monitoring and evaluation activities to ensure high quality of IRS operations as a pre-condition for achieving a fast and sustained reduction of vector densities. This will continue to be important during the maintenance phase of VL elimination in the Indian subcontinent. Further research is needed to determine other suitable vector control option(s) when the case numbers are very low.
